# Evaluation of a proactive outreach strategy for tobacco treatment by a student-run clinic for Asian Americans: A retrospective observational study

**DOI:** 10.18332/tpc/217701

**Published:** 2026-07-10

**Authors:** Conan H. Lee, Ryan Lin, Jingyanshan Li, Samantha Wong, Ronald Jan, Elisa K. Tong

**Affiliations:** 1 School of Medicine University of California, Davis Sacramento California United States; 2 School of Medicine University of Pittsburgh Pittsburgh United States; 3 Paul Hom Asian Health Clinic Sacramento United States

**Keywords:** Asian American, disparity, smoking cessation, smokers, counseling

## Abstract

**Introduction:**

Certain Asian American subgroups have higher smoking rates than the general population, contributing to preventable mortality. This study evaluated the implementation and outcomes of a proactive tobacco treatment outreach strategy at Paul Hom Asian Clinic (PHAC), a student-run free clinic serving Asian Americans in Sacramento, California.

**Methods:**

We conducted a retrospective observational study of all active PHAC patients from August 2020 to June 2021. Volunteers used the Ask, Advise, Connect framework, which involves assessing tobacco use, advising smokers to quit, and referring them to cessation resources via the Asian Smokers’ Quitline (ASQ). Outreach calls were made in patients’ preferred languages to collect demographics, smoking status, and referral acceptance. The ASQ provided outcomes for referred patients, including counseling completion, unreachable status, or refusal. Descriptive statistics and exploratory logistic regression identified factors associated with successful contact.

**Results:**

Among 1733 patients, 194 (11.2%) had a history of tobacco use or exposure, including 162 current smokers, 27 former smokers, and 5 passive smokers, most being non-English speakers. Proactive outreach reached 23.2% of patients, with a 24.4% referral acceptance rate. Of those referred, 8 out of 11 completed counseling, 2 were unreachable, and 1 declined. Logistic regression indicated that increasing age was associated with lower odds of contact, particularly for patients aged ≥50 years. Sex and language were not significant predictors.

**Conclusions:**

Proactive outreach by student volunteers shows promise for promoting tobacco cessation among Asian American patients. Despite barriers, nearly one-quarter accepted referral, with most engaging in counseling. These findings highlight the potential for student-run clinics to deliver culturally and linguistically tailored interventions to underserved populations.

## Introduction

Smoking is the leading cause of preventable death, and certain Asian American subgroups have higher smoking rates than the general population^1-3^. In the National Health Interview Survey (2010–2018), Asian American smoking prevalence differs by gender (9% total: 13.5% men, 5% women), with the highest odds of smoking among less educated Asian immigrant men and less educated US-born women^4^. Within-group differences in smoking prevalence exist among Asian American subgroups, from 7.6% among Chinese to 20.0% among Koreans; however, gender differences account for some differences: Chinese (13.1% men, 2.9% women), Filipino (20.6% men, 7.5% women), Asian Indian (11.6% men, 3.3% women), Vietnamese (24.4% men, 7.9% women), and Korean (19.3% men, 20.4% women)^5^. The gender difference in smoking means greater attention is needed, especially for women, about secondhand smoke exposure, or passive smoking, which is a preventable health risk with over 41000 related deaths annually^1^. Asian Americans have reported experiencing secondhand smoke at rates of 38% at home and 40% at work, and less educated Asian American women report higher rates of exposure despite similar rates of smoke-free policies with their more educated counterparts^6-8^.

Free clinics provide access to health care, especially for people who are uninsured, non-citizens, or have limited English proficiency. Student-run clinics provide significant care for the community, and 140000 patients were seen annually across 208 student-run clinics in the US^9,10^. In Sacramento, Paul Hom Asian Clinic (PHAC) is the oldest existing Asian free-clinic in the US, and the student-run clinic has provided free healthcare services to patients since 1972^11^. A previous study of 109 PHAC patients, seen in the period 2017–2020 for common skin-related complaints, showed PHAC demographics included an average age of 50 years (range: 12–78 years), 56% female, 78% Asian (half being Chinese), 62% non-English speakers, 52% uninsured, and half of those with insurance had Medicaid^12^.

As the COVID-19 pandemic disrupted clinical care, PHAC students in the cardiopulmonary department implemented a proactive outreach strategy to call patients offering a referral to the Asian Smokers’ Quitline (ASQ)^13^. ASQ is a free nationwide quitline, funded by the Centers for Disease Control and Prevention, that provides free coaching in several Asian languages (Cantonese, Mandarin, Korean, and Vietnamese) and offers free nicotine patch starter kits mailed home^13,14^. ASQ has demonstrated effectiveness with its trained coaches in assisting with long-term quitting^15^. The aim of this study is to describe the implementation and evaluation of the proactive outreach strategy for tobacco treatment with PHAC patients.

## Methods

### Patient cohort identification

This was a retrospective chart review IRB-exempt study that was conducted on active patients, or patients who had visited PHAC in the past 2 years, starting in 2020. Data were collected for tobacco status (current, former, passive, never) documented at the initial intake of the patients’ first visit to the clinic. If the patient did not have an initial tobacco status, we attempted to look at information in 2 subsequent visit notes; 420 of 1733 (24.2%) patients were missing tobacco status from their initial intake and subsequent visit notes. During the COVID-19 pandemic, clinic operations were paused for student safety, and routine intake was not conducted.

### Proactive outreach program development and implementation

In July 2020, undergraduate and medical student volunteers at PHAC developed the proactive outreach strategy with a faculty mentor, who leads the local health department’s tobacco coalition cessation subcommittee. Students were trained in the Ask, Advise, and Connect framework to assess the patient’s current tobacco use, offer a referral to ASQ for free nicotine patches and one-on-one telephone counseling, and submit a quitline referral so the patient receives a call from ASQ^16^. This workflow was documented in a protocol so future volunteers at PHAC can continue to refer patients. During these calls, volunteers would confirm important patient demographic information using questionnaires, such as sex, age, and preferred language. The patient’s current tobacco use was also recorded as current, former, and passive smoking. Finally, a successful outcome of the call was also recorded, including successful patient contact or successful referral to ASQ.

Patient outreach was conducted throughout August 2020 to June 2021. Outreach calls were made to the patient in their preferred language via a student interpreter. The Asian language services provided by PHAC primarily are Chinese (Cantonese, Mandarin, Taishanese) and Vietnamese, but also include Korean and Hmong. For languages not offered by PHAC, attempted calls were conducted in English. If a patient had a family member who could speak English, the family member was asked to assist as an interpreter. If a patient could not be reached after three phone calls, a voicemail was left asking them to contact PHAC if they needed any healthcare services. Voicemails were not left if the phone number was disconnected. Since the outreach was initiated during the pandemic, patients were not called through the clinic phone number. Instead, volunteers called anonymously from their own phone numbers so that the patients would only see ‘Unknown Caller ID’. Student volunteers who called introduced themselves as PHAC team members, asked patients to confirm their identity, and informed them that their tobacco status had been identified in their PHAC charts. Patients who stated they were never smokers and were incorrectly classified were removed from the PHAC dataset.

### Statistical analysis

Descriptive statistics are reported as frequencies and percentages for categorical variables and as means ± standard deviation (SD) for continuous variables. Demographic variables included sex (male, female), age (continuous in years; also categorized as <50 vs ≥50 years), and primary language (English, Chinese, Vietnamese, etc.). Age was treated as a continuous variable in regression models, with a categorical version used in a secondary analysis. Chi-squared and Fisher’s exact tests were used for categorical variables, while t-tests were used for continuous variables, as appropriate. Comparisons among current, former, and passive smokers were adjusted using the Benjamini-Hochberg method to control for false discovery rate. An exploratory multivariable logistic regression was performed to identify factors associated with being contacted by PHAC volunteers among patients identified in the chart review, adjusting for sex, age, and language. A second regression model examined successful patient contact using the same variables, but with age categorized. Statistical significance was defined as p<0.05. All analyses were performed using Stata 18.

## Results

### Characteristics of PHAC patients with tobacco use or exposure

**Figure 1:**
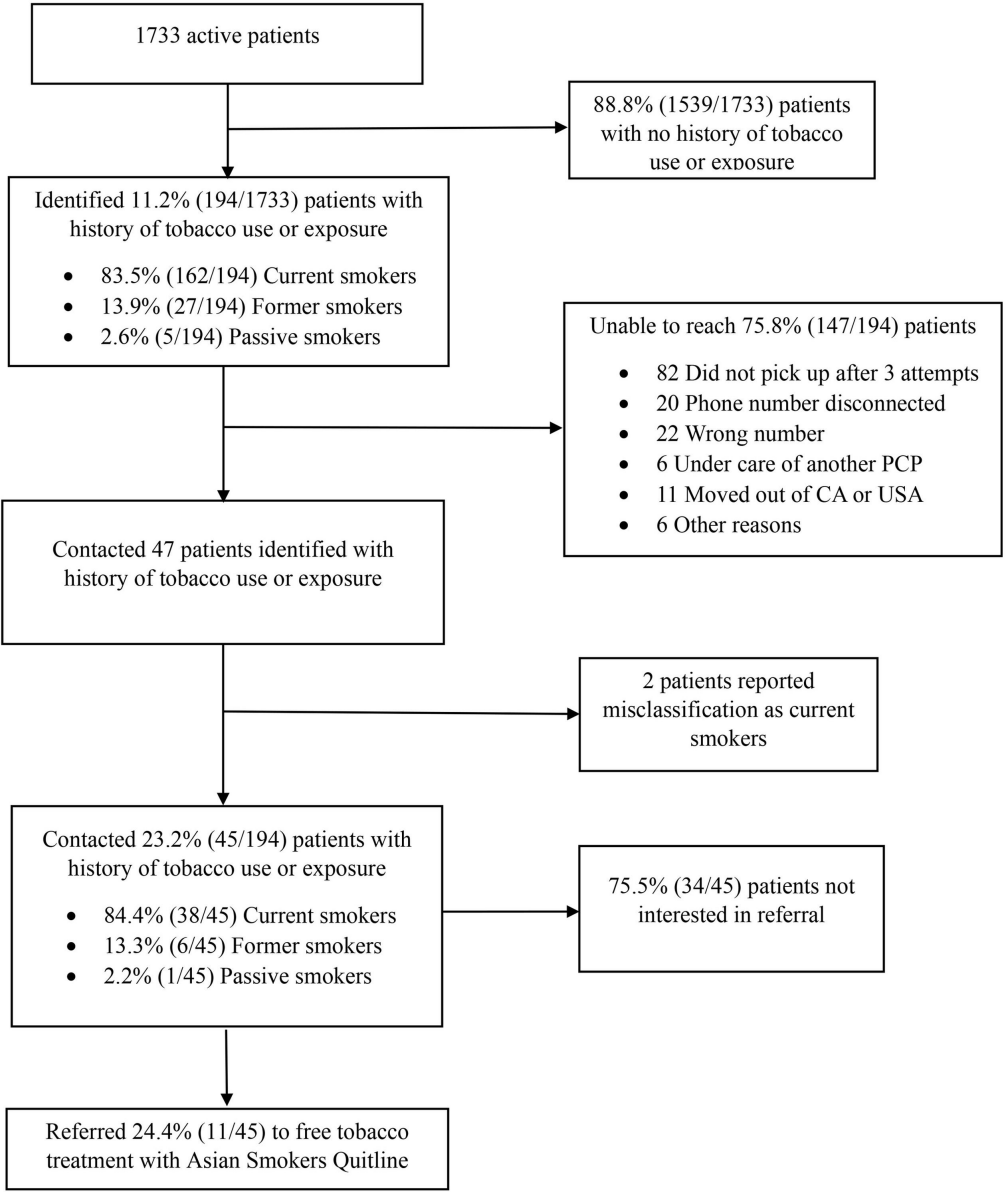
Diagram of proactive outreach with contact rates and outcomes among PHAC patients between August 2020 and June 2021

A total of 1733 active PHAC patients were identified, among them 194 patients were identified with a history of tobacco use or exposure (Figure 1). Of the 194 patients, 83.5% were current smokers, 13.9% were former smokers, and 2.6% were passive smokers. Among active PHAC patients, the PHAC current smoking prevalence was 9.3%, and any smoking history or exposure was 11.2%. Current and former smokers had similar demographics, with a mean age of >50 years, the majority being men, and about half being Chinese speakers (Table 1). The other non-English languages (13%) were languages not typically serviced at PHAC (Mongolian, Tagalog, Laotian) and non-Asian languages (Farsi, Russian, Spanish). There were statistically significant differences between sex and smoking status, with higher proportions of males being current or former smokers (Table 1). Furthermore, the majority of patients with tobacco use or exposure were male, compared to those with no tobacco use or exposure (Table 2). Among the few passive smokers, the patients were all female and mostly Chinese-speaking, except for one Mongolian-speaking patient.

PHAC: Paul Hom Asian Clinic. PCP: primary care physician. CA: California.

**Table 1:** Characteristics of PHAC patients with history of tobacco use or exposure and contacted during the COVID pandemic, August 2020 – June 2021 (*N*=194)

Characteristics	Patients with tobacco use or exposure			p	Patients contacted			p
	Current (N=162) n (%)	Former (N=27) n (%)	Passive (N=5) n (%)		Current (N=38) n (%)	Former (N=6) n (%)	Passive (N=1) n (%)	
**Age** (years), mean (SD)	53.37 (13.21)	55.67 (12.84)	52.00 (13.11)	0.675	50.45 (13.67)	49.00 (16.98)	54.00	0.940
**Age** (years)								
18–24	1 (0.6)	1 (3.7)	0	0.584	1 (2.6)	1 (16.7)	0	0.586
25–44	42 (25.9)	5 (18.5)	1 (20)		12 (31.6)	2 (33.3)	0	
45–64	84 (51.9)	13 (48.1)	3 (60)		19 (50)	2 (33.3)	1 (100)	
≥65	35 (21.6)	8 (29.6)	1 (20)		6 (15.8)	1 (16.7)	0	
**Sex**								
Male	134 (82.7)	21 (77.8)	0	**<0.001**	32 (84.2)	5 (83.3)	0	0.191
Female	28 (17.3)	6 (17.3)	5 (100)		6 (15.8)	1 (15.8)	1 (100)	
**Primary language group**								
Chinese^a^	80 (49.4)	13 (48.1)	4 (80)	0.805	17 (44.7)	4 (66.7)	1 (100)	0.957
Vietnamese	33 (20.4)	6 (22.2)	0		11 (28.9)	1 (16.7)	0	
Korean	1 (0.6)	0	0		0	0	0	
Hmong	2 (1.2)	1 (3.7)	0		1 (2.6)	0	0	
English	24 (14.8)	4 (14.8)	0		6 (15.8)	1 (16.7)	0	
Mongolian	12 (7.4)	1 (3.7)	1 (20)		1 (2.6)	0	0	
Tagalog	1 (0.6)	1 (3.7)	0		1 (2.6)	0	0	
Laotian	1 (0.6)	0	0		0	0	0	
Farsi	1 (0.6)	0	0		0	0	0	
Spanish	5 (3.1)	1 (3.7)	0		0	0	0	
Russian	2 (1.2)	0	0		1 (2.6)	0	0	

a Chinese languages including Cantonese, Mandarin, and Taishanese.

**Table 2:** Descriptive summary of characteristics of the proportion of PHAC patients with a history of tobacco use or exposure and no history of tobacco use or exposure (*N*=1733)

Characteristics	No history of tobacco use or exposure (N=1539) n (%)	History of tobacco use or exposure (N=194) n (%)	p
**Age** (years), mean (SD)	57.57 (16.7)	57.51 (13.1)	0.943
**Sex**			
Female	980 (64.6)	39 (20.1)	**<0.001**
Male	537 (35.4)	155 (79.9)	
**Primary language group**			
Chinese^a^	673 (43.7)	97 (50.0)	0.452
Vietnamese	322 (20.9)	39 (20.1)	
Korean	9 (0.6)	1 (0.5)	
Hmong	73 (4.7)	3 (1.6)	
English	349 (22.7)	28 (14.4)	
Mongolian	41 (2.7)	14 (7.2)	
Tagalog	10 (0.7)	1 (0.5)	
Laotian	0 (0)	1 (0.5)	
Farsi	3 (0.2)	1 (0.5)	
Spanish	26 (1.7)	6 (3.1)	
Russian	2 (0.1)	2 (1.0)	
Other/unknown	31 (2.0)	0 (0)	

a Chinese languages including Cantonese, Mandarin, and Taishanese.

### PHAC outreach contact rates

Among the 194 patients with a history of tobacco use or exposure, 23.2%(45/194) were contacted by PHAC volunteers; 2 patients who were contacted stated they were never smokers and were removed from the PHAC dataset. Of the 75.8% of patients (147/194) who were unable to be contacted, 82 (55.8%) did not answer after three attempts, 20 (13.6%) had disconnected numbers, and 22 (15.0%) had incorrect numbers. Additionally, 6 (4.1%) were under the care of another primary care provider, 11 (7.5%) had moved out of California or the United States, and 6 (4.1%) could not be reached for other miscellaneous reasons (Figure 1). Among the 45 patients contacted, 84.4% were current smokers, 13.3% former smokers, and 2.2% were passive smokers. The current and former smokers contacted were similar, with a mean age of about 50 years, the majority being men who mostly spoke Asian languages such as Chinese and Vietnamese, served by PHAC (Table 1).

### ASQ referrals and service outcome

For ASQ referrals, 11 patients consented to be called. Among the 11 patients referred, all were current smokers, male, and spoke an Asian language (6 Chinese, 5 Vietnamese). ASQ service outcomes demonstrated that 72.7% of the referred patients received counseling, but 2 were unable to be contacted, and 1 declined assistance. There were no significant differences between those who refused a referral (n=34) and those who were referred (n=11), although all referred patients were male (Table 3).

**Table 3:** Comparison of statistics between PHAC patients who were referred and not referred

Characteristics	Not referred (N=34) n (%)	Referred (N=11) n (%)	p
**Age** (years), mean (SD)	49.09 (13.44)	54.18 (14.85)	0.293
**Sex**			0.168
Female	7 (20.6)	0 (0)	
Male	27 (79.4)	11 (100)	
**Smoking status**			0.721
Current	29 (85.3)	9 (81.8)	
Former	4 (11.8)	2 (18.2)	
Passive	1 (2.9)	0 (0)	
**Primary language group**			0.813
Chinese^a^	17 (50.0)	5 (45.5)	
Vietnamese	7 (20.6)	5 (45.5)	
Hmong	1 (2.9)	0 (0)	
English	6 (17.7)	1 (9.1)	
Mongolian	1 (2.9)	0 (0)	
Tagalog	1 (2.9)	0 (0)	
Russian	1 (2.9)	0 (0)	

a Chinese languages including Cantonese, Mandarin, and Taishanese.

In multivariable logistic regression analysis of the 194 PHAC patients with a history of tobacco use or exposure, age was the only demographic factor significantly associated with being contacted (Table 4). Increasing age was associated with lower odds of contact (adjusted odds ratio, AOR=0.96; 95% CI: 0.94–0.99; p=0.01). Male sex was not significantly associated with contact compared with female sex (AOR=1.62; 95% CI: 0.61–4.32; p=0.33). Similarly, language preference was not significantly associated with contact status, although there was a trend toward higher odds of contact among Vietnamese-speaking patients compared with English-speaking patients (AOR=2.59; 95% CI: 0.88–7.46; p=0.08). No significant association was observed for Chinese-speaking patients (AOR=1.63; 95% CI: 0.67–4.00; p=0.28). In a repeat multivariable logistic regression with age as a categorical variable, age ≥50 years was significantly associated with lower odds of successful patient contact (AOR=0.30; 95% CI: 0.13–0.70; p=0.005) (Table 5).

**Table 4:** Multivariable logistic regression of demographics to predict successful patient contact with age as a continuous variable. The odds ratios represent the likelihood of successfully contacting a patient based on the listed demographic characteristics (*N*=194)

Variables	AOR	95% CI	p
Age (years)	0.96	0.94–0.99	**0.01**
Male	1.62	0.61–4.32	0.33
Chinese	1.63	0.67–4.00	0.28
Vietnamese	2.59	0.88–7.46	0.08

AOR: adjusted odds ratio. Females serve as the reference group for the sex variable, and English serves as the reference group for the language variables.

**Table 5:** Multivariable logistic regression of demographics to predict successful patient contact with age as a categorical variable. The odds ratios represent the likelihood of successfully contacting a patient based on the listed demographic characteristics (*N*=194)

Variables	AOR	95% CI	p
Age ≥50 years	0.30	0.13–0.70	**0.005**
Male	1.79	0.56–5.69	0.323
Chinese	1.31	0.45–3.79	0.621
Vietnamese	2.10	0.63–7.03	0.227

AOR: adjusted odds ratio. Age <50 years serves as the reference group for the categorical age variable, Female serves as the reference group for the sex variable, and English serves as the reference group for the language variables.

## Discussion

The PHAC current smoking prevalence of almost 10% is higher than reported for California’s Asian American population tobacco prevalence of 7%, which may reflect the higher use rates in the PHAC immigrant population^8,17^. The exposure rate seems low, compared to survey data showing 15–22% of Californian Asian American women reporting smoking inside the home, and may be due to underreporting or documentation. This study describes the first effort, to our knowledge, of a student-run free clinic for Asians conducting proactive outreach for tobacco treatment with the ASQ. As the COVID pandemic stay-at-home orders disrupted clinical care, this study demonstrated the feasibility of adapting to a proactive outreach protocol offering in-language, telephone-based services in collaboration with the ASQ for providing free nicotine patches mailed to patients’ homes. Nearly a quarter of the active patients with a history of tobacco use or exposure were contacted, of whom nearly a quarter agreed to an ASQ referral, and three-quarters engaged with ASQ services. In the exploratory multivariable regression analysis, younger age was a statistically significant predictor of patients being contacted with the proactive outreach. Specifically, patients aged <50 years had statistically significantly greater odds of successful contact than patients aged ≥50 years. The proactive outreach by a trusted clinic source allowed PHAC volunteers to conduct culturally and linguistically appropriate motivational interviews and educate about free ASQ services. Counseling for mental and physical health can be stigmatized within Asian communities, so providing culturally and linguistically appropriate care and resources is important for engagement^18^.

While this proactive outreach was initiated due to the COVID pandemic disrupting clinical care, it is important to recognize that unique stressors for the Asian American community may be associated with coping behaviors of increased smoking that need to be addressed with trusted community-based sources^19^. During the COVID-19 pandemic, there was an increase in smoking prevalence among Asian Americans, with increased rates of relapse from individuals who previously quit smoking^19^. The increased racism and xenophobia towards the Asian American population during the COVID-19 pandemic were also found to be associated with increased smoking prevalence^20^. Ethnic groups, including young Asian adults, who experienced COVID-related stressors were found to have a higher risk for using both cigarettes and e-cigarettes^21^.

The PHAC patient population reflects the increasing cultural, linguistic, and socio-economic diversity of the Sacramento area and California in general. PHAC volunteers reached mostly patients speaking Chinese or Vietnamese, but were able to reach one patient each for Mongolian, Tagalog, Hmong, and Russian languages. These latter group of patients all declined referrals to the quitline, and, although the reason for declining was not asked, the lack of language services in the program may have deterred their interest. According to the US Census from 2018 to 2022, almost one quarter of the Sacramento population was born in a foreign country, and over one third of families spoke a language other than English in their household^22^. Chinese dialects constitute the largest Asian language group in California, with over one million speakers, with Tagalog coming in second with over six hundred thousand speakers^17^. Half of Vietnamese and Mongolian Americans (as well as Burmese) have limited English proficiency, and Mongolian and Hmong Americans have the highest poverty rates at just below one-quarter of the population^17^. Korean, Cambodian, Thai, and Indonesian Americans have the highest uninsured rates at just under 10%^17^. Community-based outreach is needed for these vulnerable Asian American and other non-English speaking subgroups.

The contact and referral rates in this study with PHAC patients show both similarities and differences compared to other proactive outreach efforts with non-English-speaking patients. In Los Angeles County during the COVID pandemic, over 600 English- and Spanish-speaking Medicaid patients were contacted to offer Kick It California referrals; the contact rate was over 50% (higher for Spanish speakers), but the referral rate was 30% (lower for Spanish speakers), likely aided by the use of a local identifiable phone number^23^. In Minnesota (2018–2019), over 300 Vietnamese-speaking patients were mailed invitations with follow-up calls; recruitment and referral rates were 25% and 17%, respectively, with higher rates among those receiving motivational interviewing versus interactive voice response^24^. Compared to these studies, the PHAC outreach demonstrates similar contact and referral rates, supporting the feasibility and acceptability of population-based outreach offering free in-language quitline services.

From 2012 to 2019, in its first 7 years, ASQ has been used by more than fourteen thousand Asian-speaking smokers across the US, and the main referral sources were mostly media, followed by family and friends; healthcare providers have been a smaller percentage, but have the potential to be a sustainable referral source^13^. Previous community-based promotion of ASQ at shopping centers was labor-intensive, and press releases to ethnic media and direct-to-member mailings have been more efficient^25^. California’s free quitline has been regularly promoted in quarterly statewide household mailings to seven million Medi-Cal members, including during the COVID pandemic (October 2020 to June 2021) with a free nicotine patch offer, but these have been only English/Spanish materials with no Asian-specific language promotion^21^. As ASQ has an additional twenty-dollar financial incentive for callers that launched in February 2024, PHAC volunteers are doing additional community-based outreach with Asian businesses and community organizations^25^.

Future proactive outreach should consider how to increase contact rates with Asian American patients. Younger age was associated with being more likely to be contacted, which may be related to higher levels of comfort with using technology^26^. Still, over half of those who were not contacted were those who did not pick up the telephone after 3 attempts. One major contributing factor was the method of contacting patients. PHAC volunteers called anonymously and disguised their own personal phone numbers as ‘Unknown Caller ID’. This method of calling was previously used when communicating lab results, but patients were given a reminder after visits to expect a call from an unknown number. No patients were informed prior to proactive outreach because this project was initiated during the COVID pandemic, when the clinic was closed. Patients might be asked if they agree to being contacted by text or email.

### Limitations

This study has several limitations. First, some data, including tobacco status, were self-reported, which could introduce information bias and misclassification. Chart documentation errors and social desirability bias may have further affected data accuracy. Second, the sample size was relatively small, particularly for certain subgroups, which may have limited statistical power. Third, residual confounding by unmeasured factors could not be fully addressed, and potential non-response bias may have arisen from anonymous phone calls or incomplete responses. Fourth, the study design and analyses do not allow for the establishment of causal associations. Finally, this study focused on patient engagement with evidence-based treatment at the PHAC clinic in Sacramento, which may limit generalizability to other settings, and patient responses may have been influenced by the COVID-19 pandemic and stay-at-home orders during the study period.

## Conclusions

This study shows that proactive outreach to patients in a student-run clinic for Asian Americans is feasible and acceptable for the promotion of free evidence-based tobacco treatment with ASQ. The PHAC patients with a history of tobacco use and exposure were highly diverse, including representation from underserved Asian American subgroups and other non-English speaking groups. The majority of PHAC patients who agreed to a referral engaged in ASQ services. This study underscores how proactive outreach to populations with limited English proficiency and low socio-economic status can be conducted in a resource-limited setting to engage with free tobacco treatment.
